# Bremachlorin-Based Photodynamic-Therapy-Induced Effects in Low-Oxygenated Pancreatic Ductal Adenocarcinoma (PDAC) Tumor Microenvironment

**DOI:** 10.3390/ijms27209374

**Published:** 2026-10-21

**Authors:** Roisin McMorrow, Henriette S. de Bruijn, Ivo Que, Karishma Lila, Thierry P. P. van den Bosch, Michail Doukas, Clemens W. G. M. Löwik, Laura Mezzanotte, Dominic J. Robinson

**Affiliations:** 1 Department of Radiology and Nuclear Medicine, Erasmus MC Cancer Institute, University Medical Center Rotterdam, 3015 GD Rotterdam, The Netherlandslowik.clemens57@gmail.com (C.W.G.M.L.); 2 Department of Molecular Genetics, Erasmus MC Cancer Institute, University Medical Center Rotterdam, 3015 GD Rotterdam, The Netherlands; 3 Department of Otorhinolaryngology and Head and Neck Surgery, Erasmus MC Cancer Institute, University Medical Center Rotterdam, 3015 GD Rotterdam, The Netherlands; d.robinson@erasmusmc.nl; 4 Department of Pathology, Erasmus MC Cancer Institute, University Medical Center Rotterdam, 3015 GD Rotterdam, The Netherlands; k.lila@erasmusmc.nl (K.L.); t.vandenbosch@erasmusmc.nl (T.P.P.v.d.B.); m.doukas@erasmusmc.nl (M.D.)

**Keywords:** photodynamic therapy, Bremachlorin, fluence rate, spectroscopy, tumor microenvironment, pancreatic ductal adenocarcinoma, colon adenocarcinoma

## Abstract

Photodynamic therapy (PDT) is a minimally invasive treatment whose efficacy is strongly influenced by tumor oxygenation and the tumor microenvironment (TME). Pancreatic ductal adenocarcinoma (PDAC) is characterized by severe hypoxia, dense stroma, and immune suppression, limiting therapeutic responsiveness. In this study, we investigated how Bremachlorin-based PDT remodels the TME in two murine PDAC models with distinct immune phenotypes (T-cell–high and T-cell–low) and compared them with the immunogenic MC38 colon carcinoma model. Single-fiber reflectance and fluorescence spectroscopy were used to monitor tumor physiology and photosensitizer dynamics during PDT, while multiplex immunohistochemistry enabled spatial profiling of stromal and immune components post-treatment. PDAC tumors exhibited significantly lower baseline oxygenation than MC38 tumors, which was not substantially improved by reducing the PDT fluence rate from 116 to 50 mW·cm^−2^. Nevertheless, PDT reduced collagen type I content in both PDAC models, indicating stromal remodeling. Importantly, illumination with a moderate fluence rate of 50 mW·cm^−2^ significantly increased CD8^+^ T-cell infiltration and improved the CD8^+^/neutrophil ratio in T-cell–high PDAC tumors, suggesting a shift toward a less immunosuppressive immune landscape. These findings demonstrate that Bremachlorin-PDT can modulate the PDAC tumor microenvironment and highlight the importance of immune context in shaping PDT outcomes, supporting rational optimization of PDT-based combination therapies.

## 1. Introduction

Photodynamic therapy (PDT) is a versatile treatment approach used for treating various cancer types as well as other diseases [1]. PDT relies on a photosensitizer (PS), a light-activated compound, that plays a crucial role in the therapeutic process. Upon absorbing light, the photosensitizer reacts with oxygen to generate reactive oxygen species (ROS), mainly singlet oxygen, that kills cancer cells. The efficacy of PDT is influenced by various factors, including the optical and physiological properties of the tumor itself, such as its light absorption, oxygenation, and vascular response [2]. The fluence and fluence rate at which light is delivered during PDT are critical parameters. PDT at a high fluence rate can lead to rapid oxygen depletion, which can outpace the rate of oxygen replenishment [3,4]. In contrast, a lower fluence rate can potentially mitigate oxygen depletion, whilst also enhancing the immune response [5,6,7]. PDT-induced cell death can lead to an immune response through primary or secondary necrotic cells that release intracellular molecules. These molecules are known as danger-associated molecular patterns (DAMPs) and are typically recognized by the innate immune cells that can lead to the activation of the immune system [8]. This immunogenic cell death (ICD) after PDT is shown in both clinical and preclinical settings [9,10,11,12,13,14].

Pancreatic cancer is a highly aggressive malignancy with one of the lowest survival rates, with an overall 5-year survival rate of 13% [15]. The most common form of pancreatic cancer is pancreatic ductal adenocarcinoma (PDAC) that originates in the ductal epithelium of the pancreas [16]. PDAC tumors are often characterized by poor oxygenation, which may contribute to treatment resistance in general and PDT specifically [17,18]. Furthermore, the response to various immunotherapy treatments has been particularly poor in PDAC, and the immune tumor microenvironment (TME) plays a crucial role in this [19].

Bremachlorin is a chlorin-based, second-generation PS also known as Radachlorin^®^. It specifically accumulates in tumors, is rapidly excreted from normal tissue, and alone shows low toxicity [20]. Bremachlorin-mediated PDT has been investigated in different cancer types such as colon, colorectal, lymphoma, lung, and oral squamous cell carcinomas [20,21,22,23,24,25]. Previously, we have shown that combining Bremachlorin-mediated PDT with immunotherapy enhances treatment response in MC38, a colon tumor that is considered a highly immunogenic tumor type [22,25]. In a subsequent study, we used two murine PDAC clones derived from KPC tumors that, in immunocompetent mice, generate tumors that differ in the TME regarding the level of T-cell infiltration, referred to as T-cell–high and T-cell–low tumors [26,27]. Employing these models, we showed an additive effect of anti-PD-L1 to PDT using Bremachlorin for the T-cell–high clone [27]. However, the lack of a synergistic effect implied that immune suppression was still present in the TME.

In the current study, our aim is to investigate the effects of Bremachlorin PDT on the TME of the T-cell–high and T-cell–low murine PDAC’s and the murine colon MC38 tumor [25,26]. Single-fiber reflectance and fluorescence spectroscopy were employed to monitor key physiological and photosensitizer parameters, including oxygen saturation (StO_2_), blood volume fraction (BVF). This was done both before and during (fluorescence photobleaching) PDT, thereby providing critical insights into the tumor’s response to treatment. Furthermore, we investigated the local immune responses to PDT by mapping the spatial distribution after PDT of immune cells within the TME. In addition, we investigated if reducing the fluence rate from the commonly used fluence rate of 116 mW·cm^−2^ for Bremachlorin-PDT to a more clinically used fluence rate of 50 mW·cm^−2^ can enhance treatment efficacy as well as inducing a more diverse and robust immune response.

## 2. Results

### 2.1. T-Cell–High and MC38 Show Response to Photodynamic Therapy

**Figure 1:**
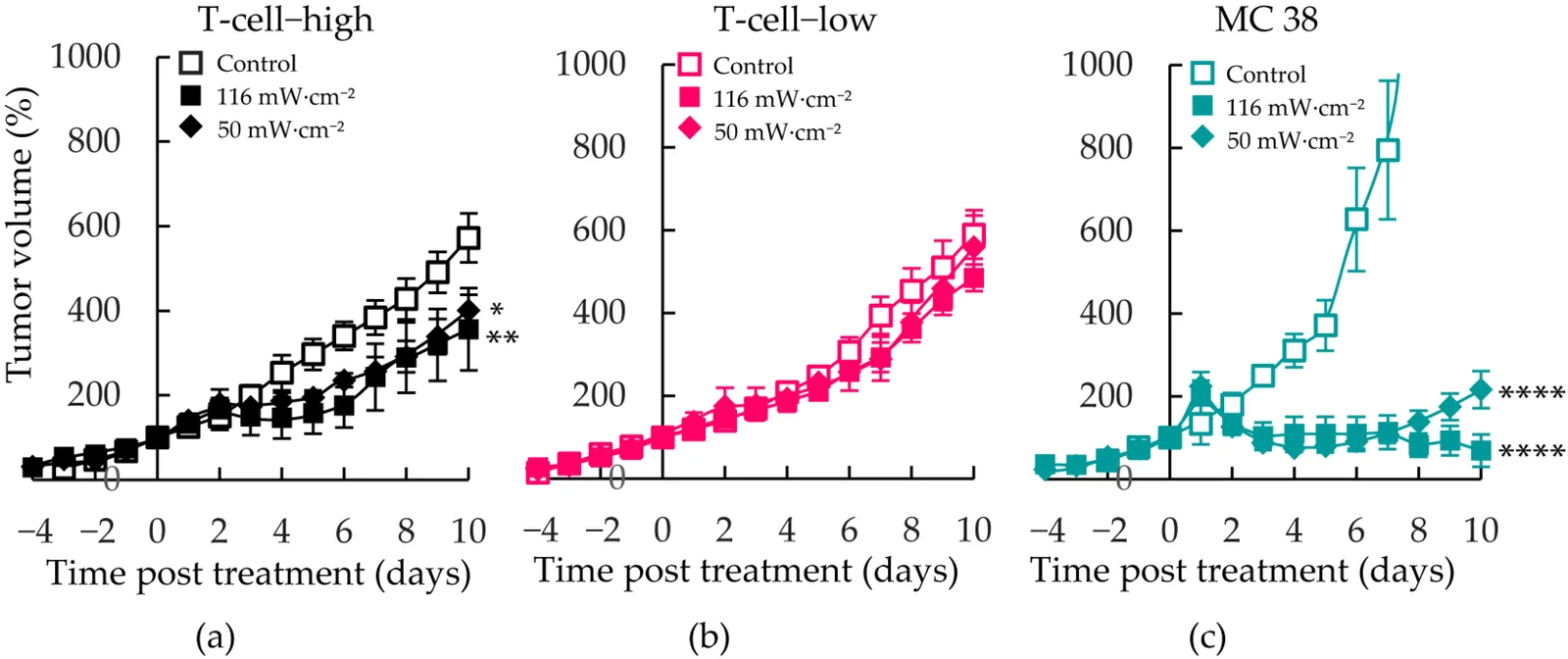
Tumor growth curves of: (**a**) T-cell–high PDAC; (**b**) T-cell–low PDAC and (**c**) MC38 tumors for control (outlined squares) or after Bremachlorin PDT to 116 J·cm^−2^ at 116 mW·cm^−2^ (filled squares) or at 50 mW·cm^−2^ (filled diamonds). Data are presented as mean ± SEM. * *p* ≤ 0.05, ** *p* ≤ 0.01, **** *p* ≤ 0.0001 compared to untreated controls.

The mean treatment volume of the tumors was 74.4 mm^3^ (range 61–97 mm^3^) and was not significantly different between groups. After 10 days of growth, the volumes of untreated T-cell–high and T-cell–low tumors reached 422 ± 121 mm^3^ and 426 ± 100 mm^3^, respectively. Individual growth curves are depicted in Supplementary Figure S1. Mean growth curves normalized to 100% at day of treatment are shown in Figure 1. The untreated MC38 tumor showed faster growth than the PDAC tumors and reached humane end points prior to day 10.

PDT using Bremachlorin to 116 J·cm^−2^ at 116 mW·cm^−2^ or 50 mW·cm^−2^ led to significant growth delay in T-cell–high tumors (*p* ≤ 0.01 and ≤0.05) but not in T-cell–low tumors 10 days after treatment. PDT of the MC38 led to a reduction in tumor volume that remained below treatment volume till the end of the study, 10 days post-treatment with 116 mW·cm^−2^ (*p* ≤ 0.0001 compared to control). Treatment with 50 mW·cm^−2^ resulted in similar results for the first couple of days, but regrowth was observed from day 8 after PDT (*p* ≤ 0.0001 compared to control). Notably, the tumor volume at day 1 after treatment was larger for MC38 tumors and not for the PDAC tumors.

### 2.2. Blood Oxygen Saturation and Blood Volume Fraction

**Figure 2:**
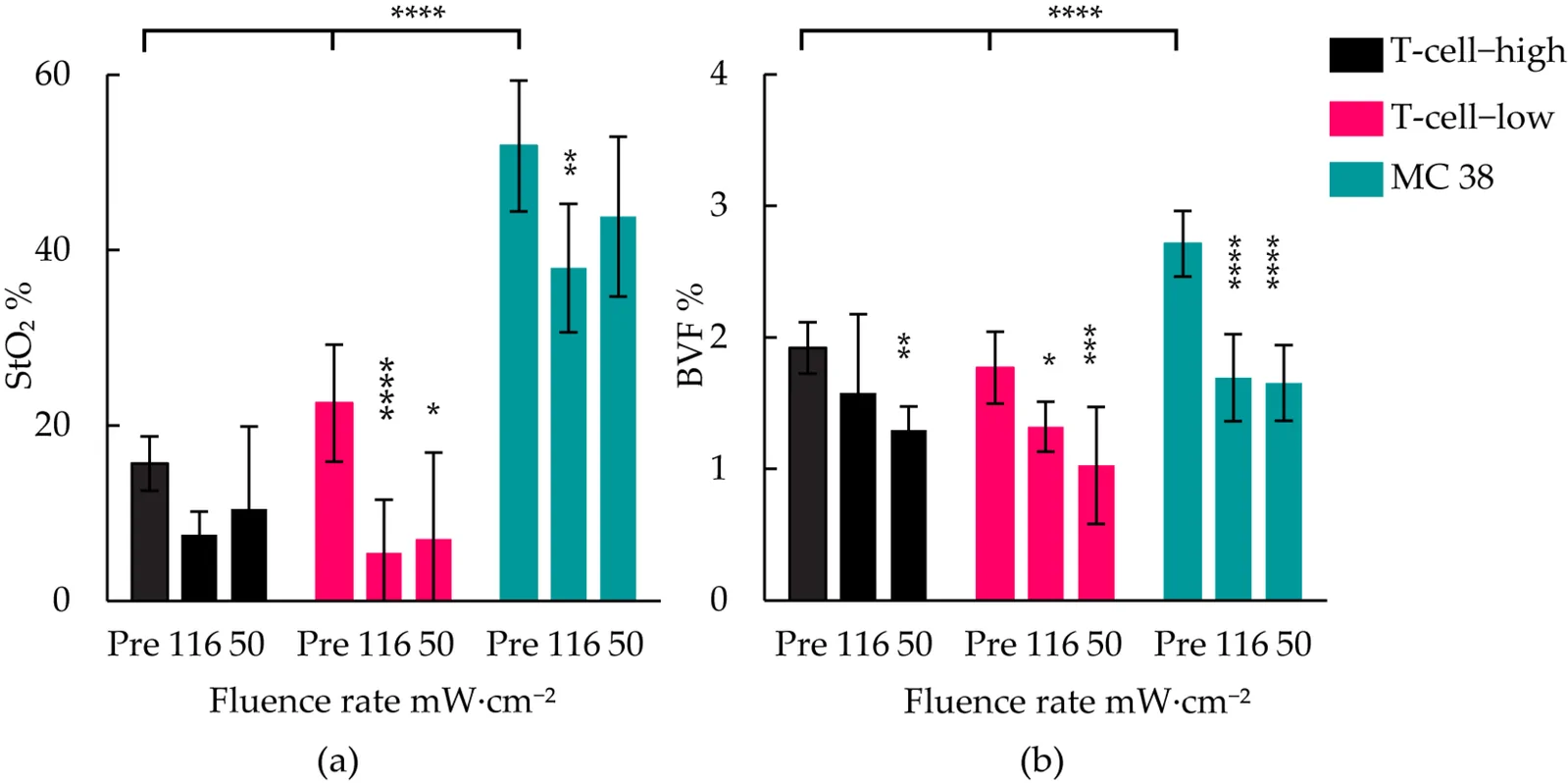
Single fiber spectroscopy data; (**a**) blood saturation (StO_2_) and; (**b**) blood volume fraction (BVF) pre– and post–photodynamic therapy using Bremachlorin to 116 J·cm^−2^ at 116 mW·cm^−2^ or 50 mW·cm^−2^. Data are presented as weighted mean ± SD. * *p* ≤ 0.05, ** *p* ≤ 0.01, *** *p* ≤ 0.001, and **** *p* ≤ 0.0001 compared between tumor types and before (pre) and after treatment.

The reflectance spectra collected during illumination were used to obtain values for blood saturation (StO_2_) and blood volume fraction (BVF). A representative reflectance spectrum and the corresponding fitting and spectral decomposition results are shown in the Supplementary Figure S2. The StO_2_ for the two PDAC tumors, i.e., T-cell–high and T-cell–low cell lines, at the start of PDT were significantly lower compared to MC38 tumors (15.7 ± 3.1 and 22.5 ± 6.7 vs. 51.9 ± 7.5%, respectively *p* ≤ 0.0001, Figure 2a). After PDT, using 116 mW·cm^−2^, the StO_2_ decreased in all tumor types to 7.5 ± 2.7, 5.5 ± 6.1 and 38.0 ± 7.3%, respectively, but was only significant for T-cell–low and MC38 tumor with *p* ≤ 0.0001 and *p* ≤ 0.01. PDT using the lower fluence rate of 50 mW·cm^−2^ also led to a decrease in StO_2_ to 10.4 ± 9.4, 7.1 ± 9.9 and 43.8 ± 9.1%, respectively, and was only significant for T-cell–low tumors with *p* ≤ 0.05. The mean level of oxygen depletion was not statistically significantly different between illumination with 116 or 50 mW·cm^−2^.

Next to the blood saturation level, also the blood volume fraction (BVF) was lower in the two PDAC tumor lines compared to the MC38 tumor (1.92 ± 0.20 and 1.77 ± 0.27 vs. 2.71 ± 0.25, respectively *p* ≤ 0.0001) (Figure 2b). After PDT, using 116 mW·cm^−2^, the BVF decreased to 1.58 ± 0.60, 1.32 ± 0.19 and 1.65 ± 0.29, respectively, which was statistically significant for the T-cell–low and MC38 tumors (*p* ≤ 0.05 and *p* ≤ 0.0001, respectively). PDT using the lower fluence rate of 50 mW·cm^−2^ led to a significant decrease in BVF in all tumor types to 1.29 ± 0.18, 1.02 ± 0.44 and 1.69 ± 0.33 (*p* ≤ 0.01, *p* ≤ 0.001 and *p* ≤ 0.0001, respectively). Both PDT treatment regimes, applying a fluence rate of either 116 mW·cm^−2^ or 50 mW·cm^−2^, led to a similar decrease in BVF.

### 2.3. Bremachlorin Photobleaching During PDT

**Figure 3:**
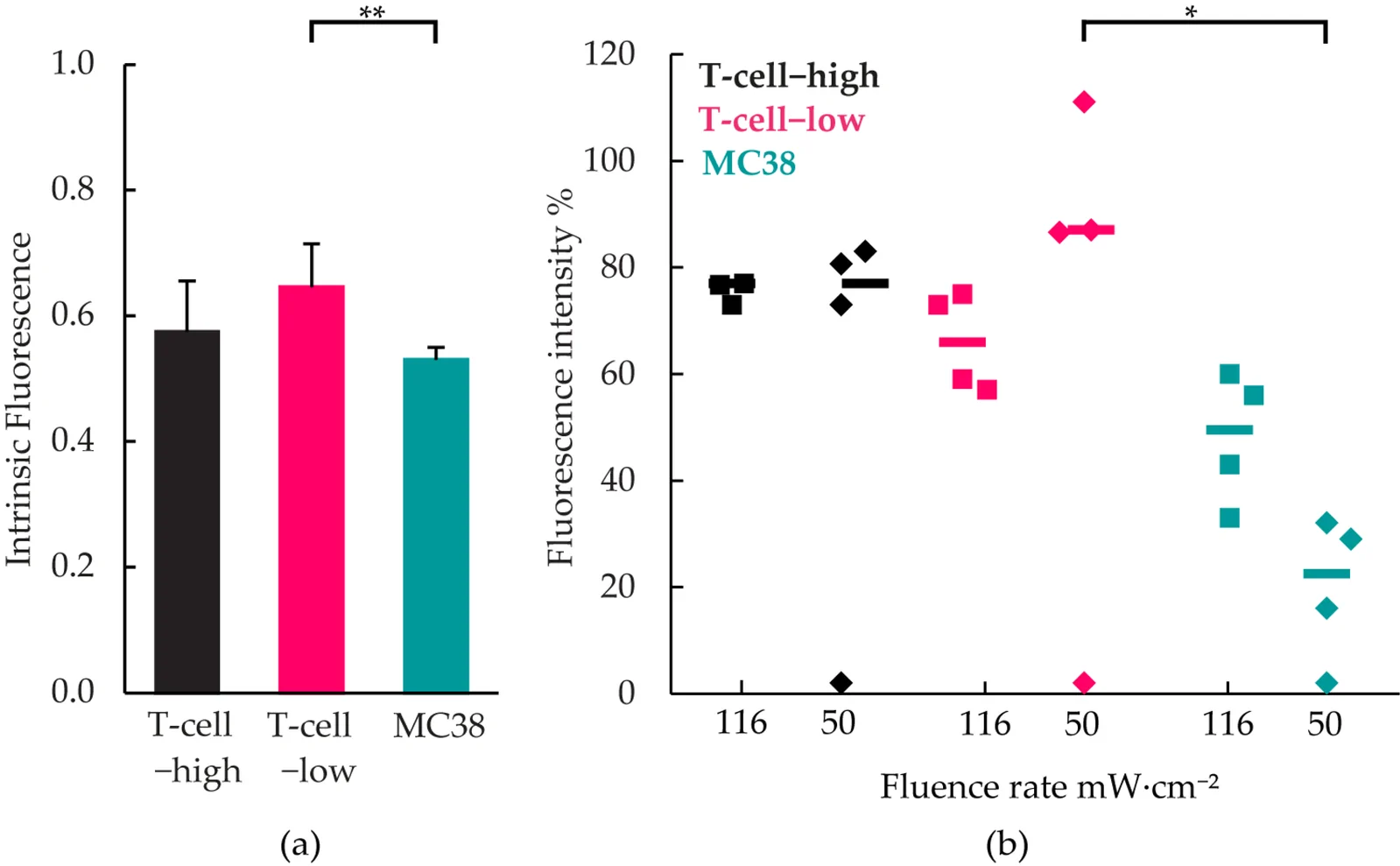
Intrinsic fluorescence intensity of Bremachlorin 6 h after administration in (**a**) T-cell–high, T-cell–low, and MC38 tumors; and (**b**) individual (dots) and group median (lines) fluorescence intensity after illumination with either 116 mW·cm^−2^ (squares) or 50 mW·cm^−2^ (diamonds) to 116 J·cm^−2^. ** *p* ≤ 0.01, * *p* ≤ 0.05.

The fluorescence spectra collected before and during illumination were used together with the data from the reflectance measurements to obtain the intrinsic fluorescence intensity, as described previously [23]. A representative fluorescence spectrum and the corresponding fitting and spectral decomposition results is shown in the Supplementary Figure S3. The intrinsic fluorescence intensity of Bremachlorin at 6 h after administration, i.e., the start of PDT, in T-cell–high, T-cell–low, and MC38 tumors was found to be 0.57 × 10^−3^ ± 0.08 × 10^−3^, 0.65 × 10^−3^ ± 0.07 × 10^−3^ and 0.53 × 10^−3^ ± 0.02 × 10^−3^ mm^−1^ nm^−1^, respectively. Only T-cell–low tumors showed significantly higher fluorescence compared to MC38 tumors (*p* ≤ 0.01) (Figure 3a).

PDT treatment led to variable levels of photobleaching of Bremachlorin. After PDT with 116 J·cm^−2^ at 116 mW·cm^−2^, the intrinsic Bremachlorin fluorescence was bleached to 73–77% in T-cell–high tumors compared to 57–75% in T-cell–low tumors (Figure 3b). Illumination with 50 mW·cm^−2^ led to more variable results. In 3 out of 4 T-cell–high tumors, fluorescence was bleached to 73–83% and in one tumor to 2%. The same was found for T-cell–low tumors, where fluorescence at the end of PDT was found to be 111–82% in 3 out of 4 tumors but almost completely photobleached in one tumor. Fluorescence spectra were recorded during illumination via a subcutaneously placed probe positioned in direct contact with the tumor while the mouse was under anesthesia. The anomalous photobleaching value exceeding 100% (111%) in a single animal is unlikely to reflect a biological effect. Rather, it is most plausibly explained by inadvertent repositioning of the probe during data acquisition, leading to sampling of a different tumor area with altered photosensitizer distribution.

**Figure 4:**
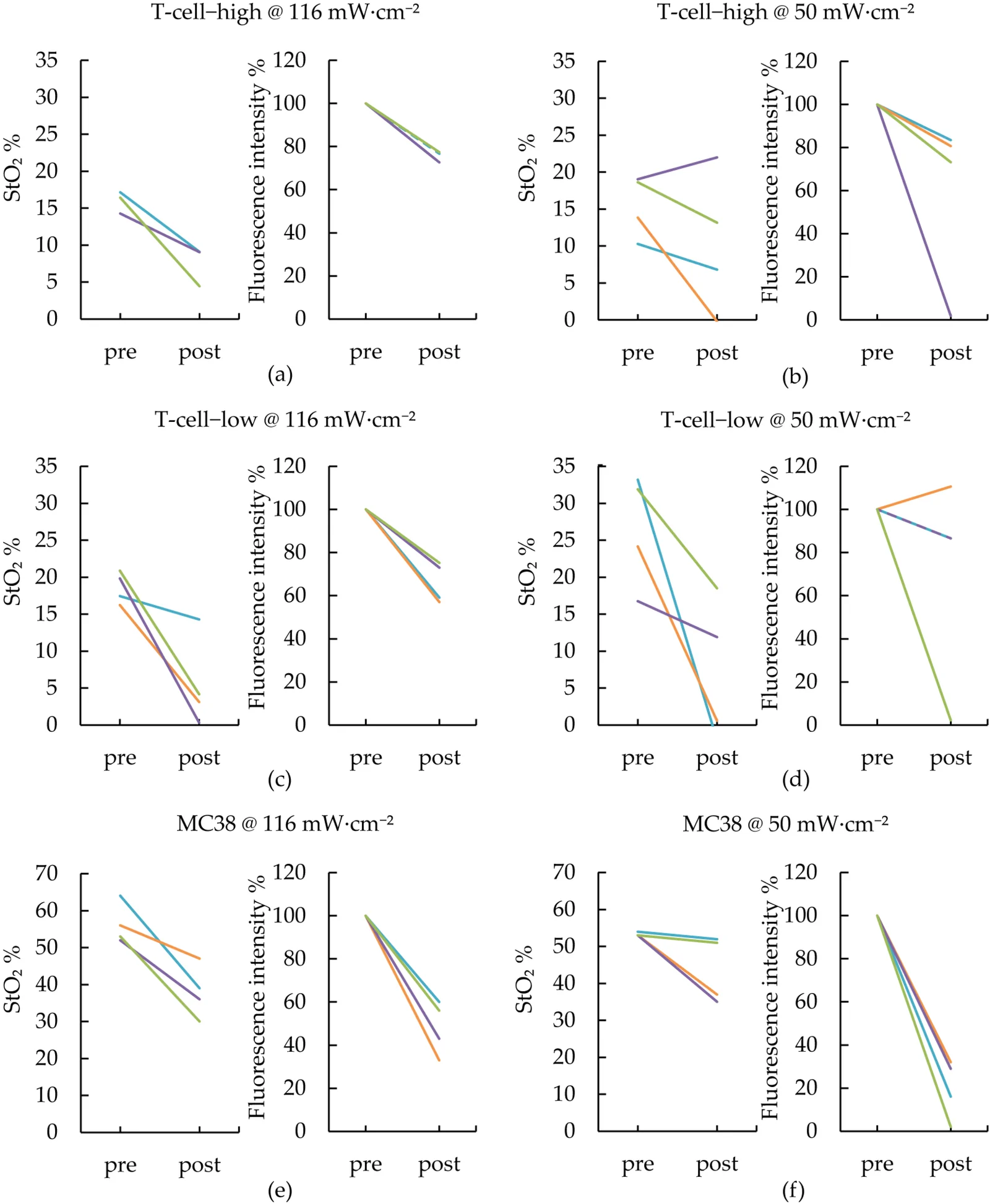
Individual blood saturation pre- and post-PDT and relative fluorescence intensity for (**a**) T-cell–high tumors and 116 mW·cm^−2^; (**b**) T-cell–high tumors and 50 mW·cm^−2^; (**c**) T-cell–low tumors and 116 mW·cm^−2^; (**d**) T-cell–low tumors and 50 mW·cm^−2^; (**e**) MC38 tumors and 116 mW·cm^−2^; (**f**) MC38 tumors and 50 mW·cm^−2^. For each experimental group, the data for StO_2_ and fluorescence intensity from an individual mouse are represented by the same colour. A line is dashed when it is overlying or close to another line for clarity reasons. Note the different *Y*-axis values for StO_2_ between PDAC and MC38 tumors.

Comparing the individual photobleaching values with the individual blood saturation values, it seems that there is a relation between the level and/or change in blood saturation level and the level of photobleaching. The two tumors that showed complete photobleaching also showed a blood saturation level that started above 20% and did not decrease below 20%, suggesting a relationship between blood saturation and photobleaching (Figure 4).

### 2.4. PDT-Treated Tumor Shows a Decrease in Ki67 and Collagen Type 1 Expression in Pancreatic Tumors

**Figure 5:**
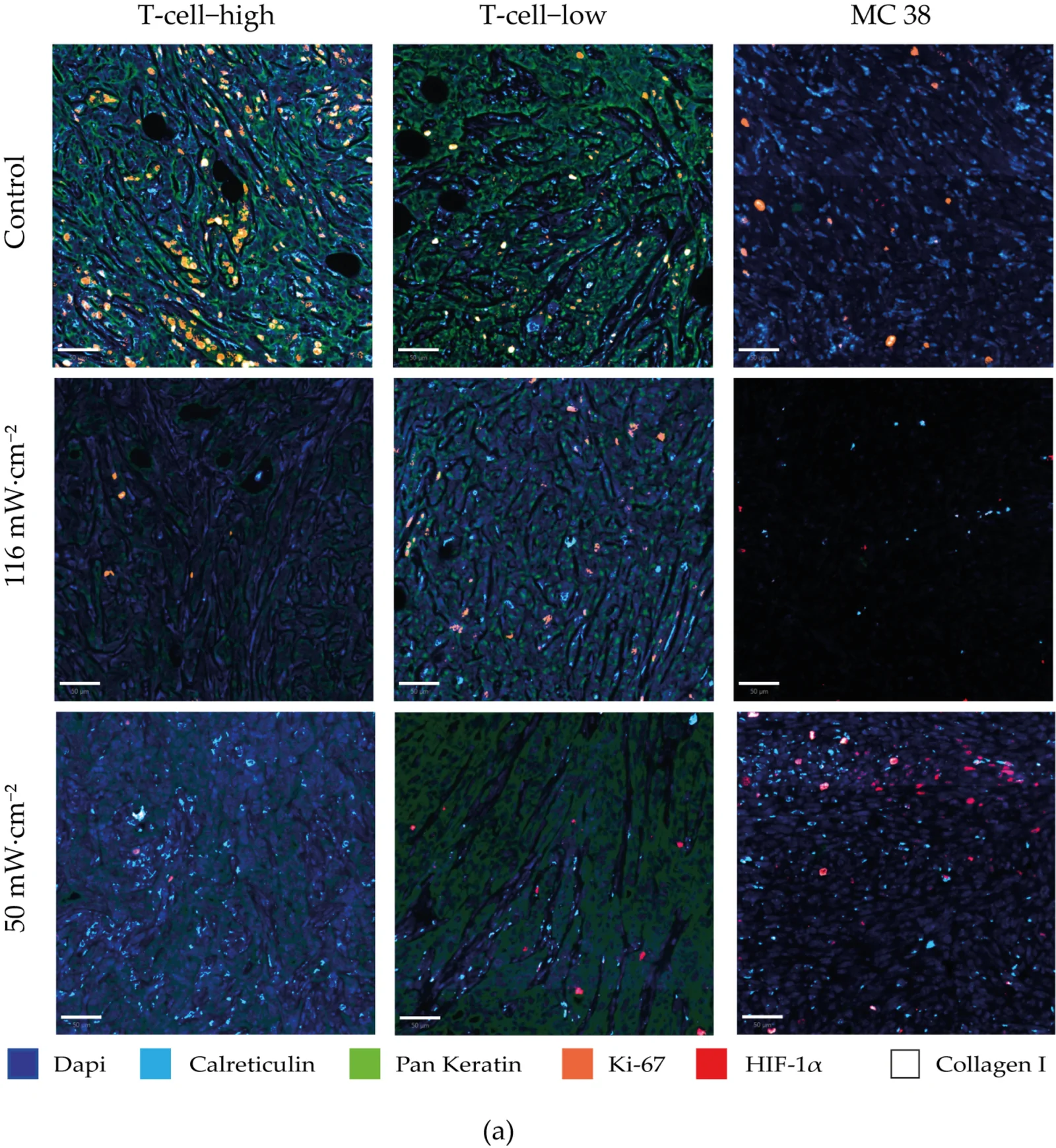

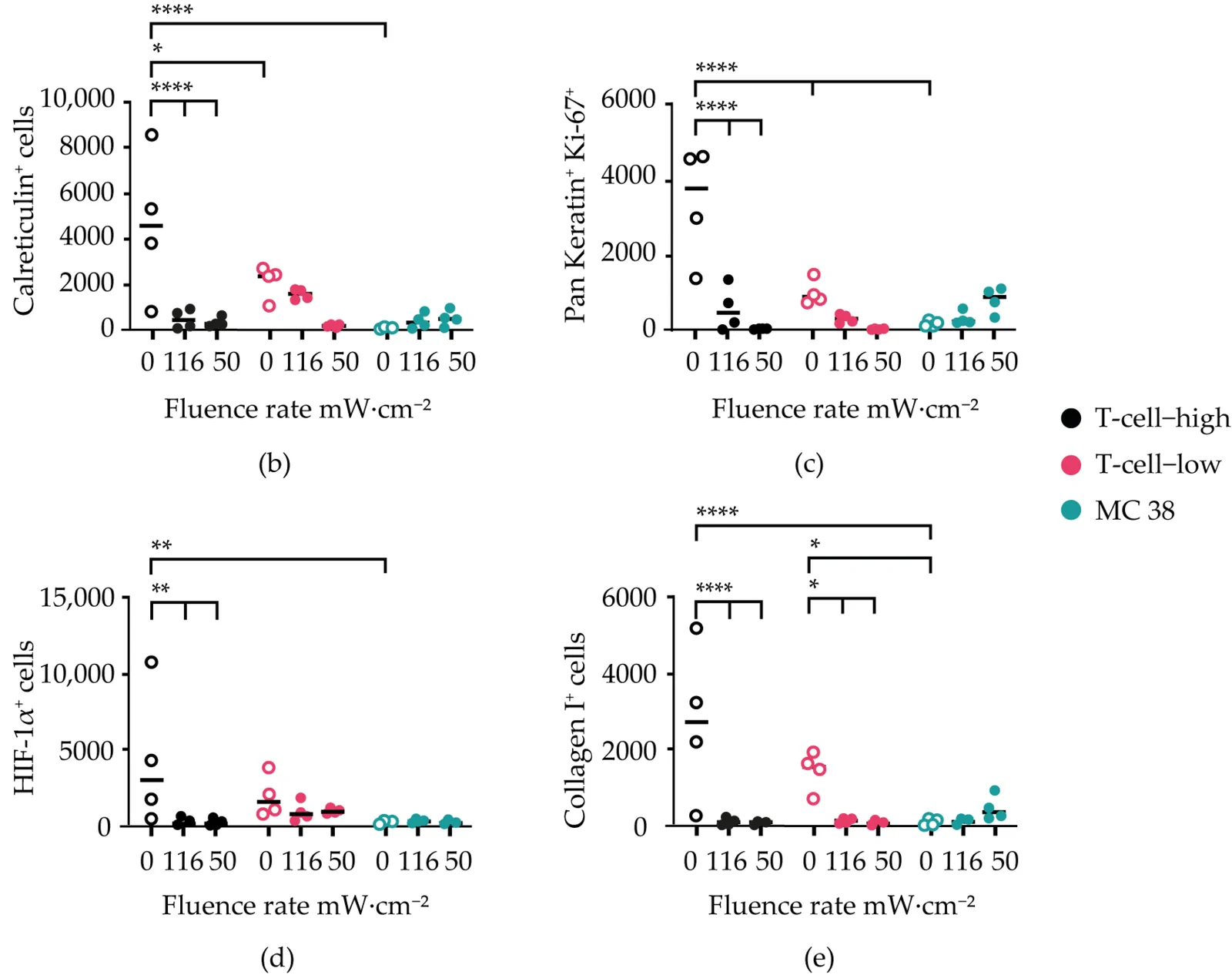
Multiplex immunohistochemistry data of DAPI, calreticulin, pan keratin, Ki-67, HIF-1α, and collagen type I of T-cell–high, T-cell–low, and MC38 untreated (outlined dots) and Bremachlorin PDT-treated tumors (116 J·cm^−2^ at 116 mW·cm^−2^ or 50 mW·cm^−2^, filled dots); (**a**) Representative fluorescent images of tissue sections; Quantification of (**b**) calreticulin^+^, (**c**) pan keratin^+^ Ki-67^+^, (**d**) HIF-1α^+^ and (**e**) collagen I^+^ cells for T-cell–high (black), T-cell–low (pink), and MC38 (green) tumor. Bar is 50 µm. Data are presented as mean ± SD. * *p* ≤ 0.05, ** *p* ≤ 0.01, **** *p* ≤ 0.0001 compared between tumor types and to pre-treatment.

Representative multiplexed fluorescent images of untreated controls and Bremachlorin PDT (116 J·cm^−2^ at 116 mW·cm^−2^ or 50 mW·cm^−2^) treated T-cell–high, T-cell–low, and MC38 tumor sections stained for calreticulin, pan keratin, Ki-67, HIF-1α, and collagen I are shown in Figure 5a. The whole tumor slices of all conditions are provided in Supplementary Figure S4. The absolute number of calreticulin^+^, pan keratin^+^ Ki67^+^, HIF-1α^+^, and Collagen I^+^ cells is shown in Figure 5b–e.

The untreated controls in PDAC and colon tumors showed differences in the expression of calreticulin, pan-keratin, and Ki-67, HIF-1α, and collagen I. Specifically, T-cell–high tumors showed higher calreticulin and co-expression of pan keratin and Ki-67 compared to T-cell–low and MC38 tumors. HIF-1α was significantly higher for T-cell–high tumors compared to MC38. Collagen I was significantly higher for the two PDAC tumors compared to MC38.

PDT treatment using a fluence rate of 116 mW·cm^−2^ or 50 mW·cm^−2^ led to a significant reduction in calreticulin expression in T-cell–high tumors compared to untreated control tumors 10 days after therapy (4.6 × 10^2^ ± 4.1 × 10^2^ and 3.2 × 10^2^ ± 2.2 × 10^2^ vs. 4.6 × 10^3^ ± 3.2 × 10^3^, respectively *p* ≤ 0.0001). In addition, the co-expression of pan keratin and Ki-67 was significantly reduced in T-cell–high tumors treated with either 116 mW·cm^−2^ or 50 mW·cm^−2^ compared to untreated control tissues (5.6 × 10^2^ ± 5.9 × 10^2^ and 22 ± 8 vs. 3.3 × 10^3^ ± 1.5 × 10^3^, *p* ≤ 0.0001). The same was observed for HIF-1α expression where the absolute number of expressing cells was reduced from 4.3 × 10^3^ ± 4.6 × 10^3^ in untreated controls to 3.1 × 10^2^ ± 2.6 × 10^2^ and 2.5 × 10^2^ ± 2.3 × 10^2^ after PDT using a fluence rate of 116 mW·cm^−2^ or 50 mW·cm^−2^, respectively (*p* ≤ 0.01). Lastly, collagen I expression was significantly reduced in both PDAC tumor lines irrespective of the fluence rate used for PDT. In T-cell–high tumors, collagen I was reduced from 2.7 × 10^3^ ± 2.1 × 10^3^ in control tumors compared to 1.2 × 10^2^ ± 96 and 86 ± 41 after PDT using a fluence rate of 116 mW·cm^−2^ or 50 mW·cm^−2^, respectively (*p* ≤ 0.0001). For T-cell–low tumors, it was reduced from 1.5 × 10^3^ ± 5.2 × 10^2^ to 1.5 × 10^2^ ± 66 and 96 ± 51, respectively (*p* ≤ 0.05).

### 2.5. Characterizing the Immune Cell Microenvironment 10 Days After PDT

**Figure 6:**
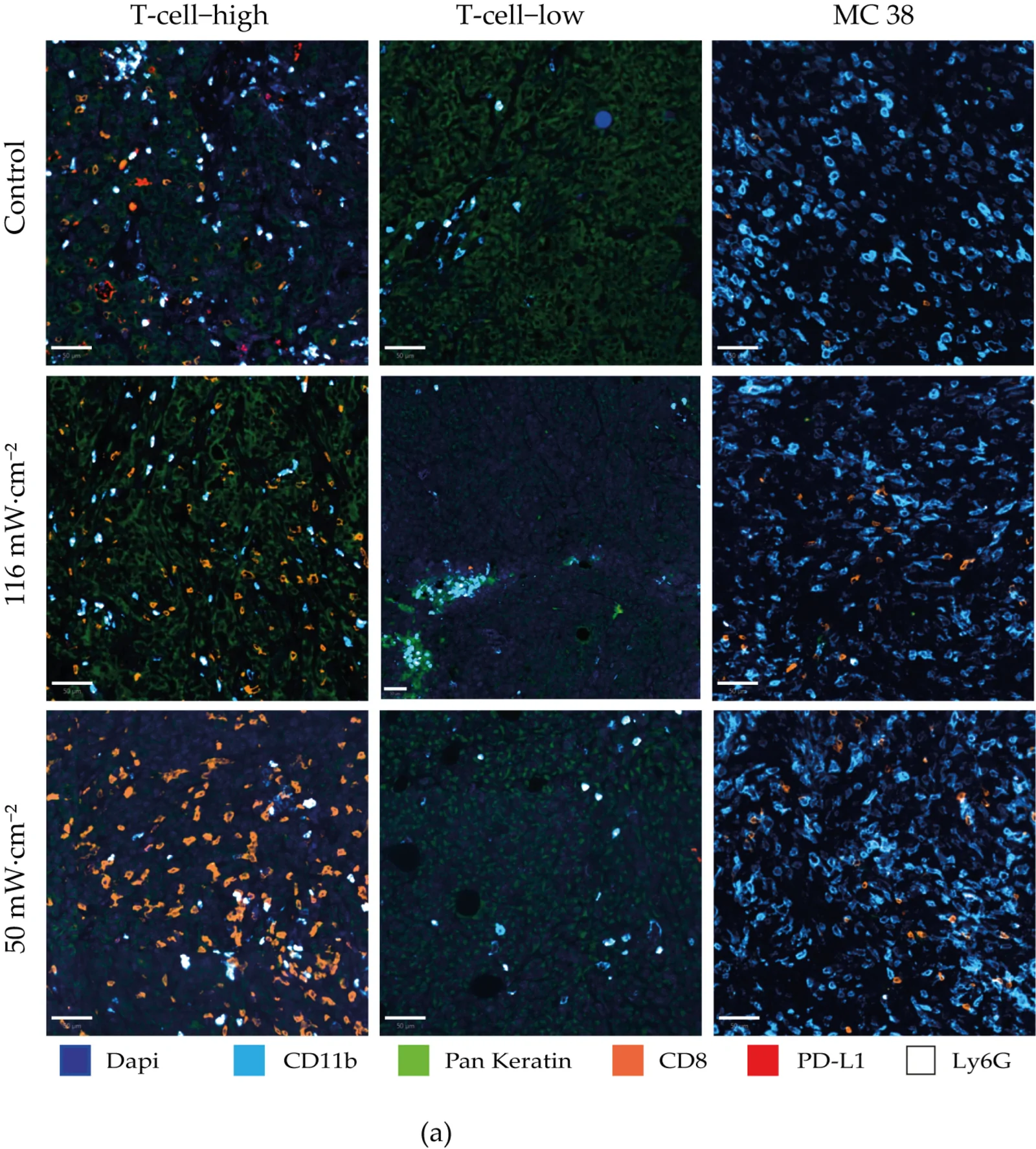

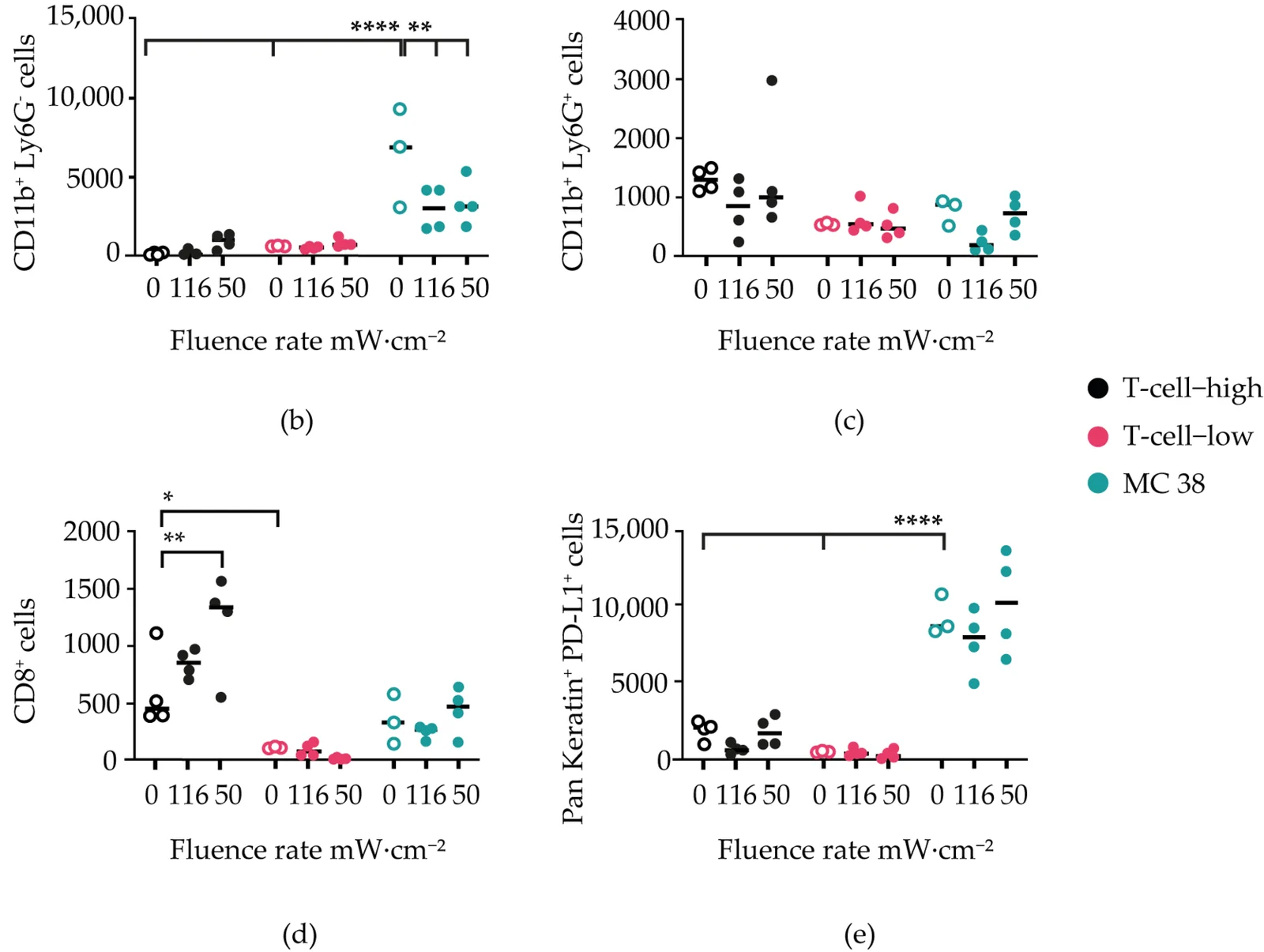
Multiplex immunohistochemistry data of DAPI, CD11b, pan keratin, CD8, PD-L1, and Ly6G of T-cell–high, T-cell–low, and MC38 untreated (outlined dots) and Bremachlorin PDT-treated tumors (116 J·cm^−2^ at 116 mW·cm^−2^ or 50 mW·cm^−2^, filled dots); (**a**) Representative fluorescent images of tissue sections; Quantification of (**b**) CD11b^+^Ly6G^−^; (**c**) CD11b^+^Ly6G^+^; (**d**) CD8^+^ and (**e**) pan keratin^+^ PD-L1^+^ cells for T-cell–high (black), T-cell–low (pink), and MC38 (green) tumors. Bar is 50 µm. Data are presented as mean ± SD. Statistical analysis was performed using two-way ANOVA followed by Boneferroni’s post hoc test for multiple comparisons, indicating significant differences at * *p* ≤ 0.05, ** *p* ≤ 0.01, **** *p* ≤ 0.0001.

Tissue sections of T-cell–high, T-cell–low, and MC38-untreated and Bremachlorin PDT (116 J·cm^−2^ at 116 mW·cm^−2^ or 50 mW·cm^−2^)–treated tumors were stained for multi-immunofluorescence analysis with CD11b, Ly6G, CD8, pan keratin, and PD-L1 (Figure 6a). The whole tumor slices of all conditions are provided in Supplementary Figure S5. The absolute number of CD11b^+^Ly6G^−^, CD11b^+^Ly6G^+^, CD8^+^, and pan keratin^+^PD-L1^+^ cells is shown in Figure 6b–e.

The untreated controls in PDAC and colon tumors showed differences in the immune cell microenvironment. Specifically, the two PDAC tumor lines show a significant lower expression of CD11b^+^ Ly6G^−^ cells compared to MC38 control tissue (both *p* ≤ 0.0001). No difference was observed in expression of CD11b^+^ Ly6G^+^ cells between the control tumors. The number of CD8^+^ cells was lowest in T-cell–low tumors and only significant compared with the T-cell–high tumors (*p* ≤ 0.05). The number of pan keratin^+^PD-L1^+^ cells was significantly higher in MC38 tumors compared to T-cell–high and T-cell–low tumors (*p* ≤ 0.0001 for both).

PDT treatment using a fluence rate of 116 mW·cm^−2^ or 50 mW·cm^−2^ led to a significant reduction in CD11b^+^Ly6G^−^-expressing cells in MC38 tumors compared to the control (3 × 10^3^ ± 1.4 × 10^3^ and 3.4 × 10^3^ ± 1.5 × 10^3^ vs. 6.4 × 10^3^ ± 3.1 × 10^3^, *p* ≤ 0.01). No significant differences were found in the number of CD11b^+^ Ly6G^+^ or pan keratin^+^PD-L1^+^ cells before PDT compared to after PDT for all tumor types, irrespective of the fluence rate used. Only CD8^+^ cells were increased in T-cell–high tumors after PDT, showing a trend in the 116 mW·cm^−2^ group and reaching statistical significance in the 50 mW·cm^−2^ group.

**Figure 7:**
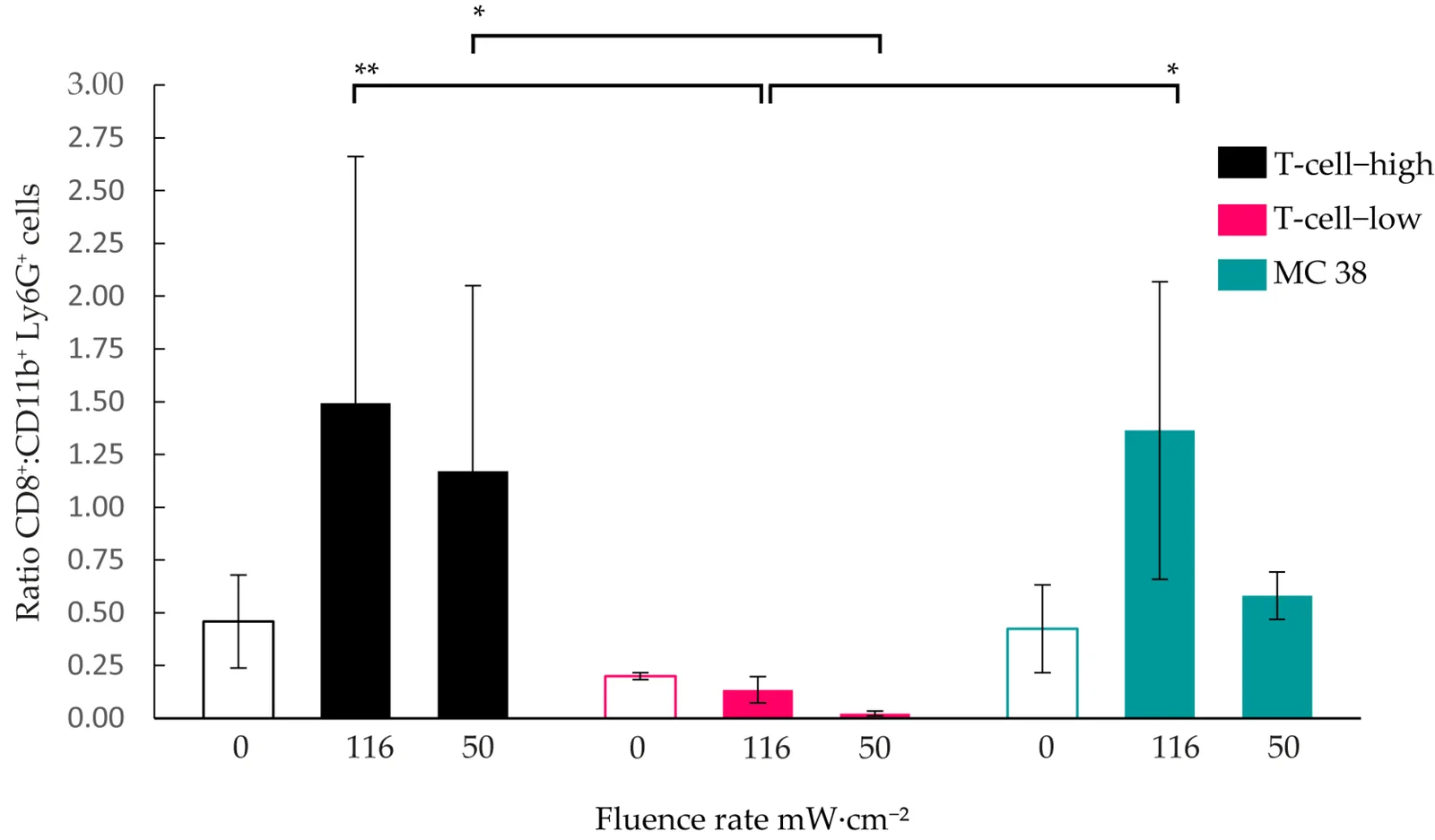
Ratio of CD8^+^ to CD11b^+^Ly6G^+^ cells from tissue sections of T-cell–high, T-cell–low, and MC38 untreated (outlined bars) and Bremachlorin PDT-treated tumors (116 J·cm^−2^ at 116 mW·cm^−2^ or 50 mW·cm^−2^, filled bars). Data are presented as mean ± SD. Statistical analysis was performed using two-way ANOVA followed by Bonferroni’s post hoc test for multiple comparisons, indicating significant differences at * *p* ≤ 0.05, ** *p* ≤ 0.01.

In addition to examining the individual immune cells, analyzing the ratios of different immune cell types can provide insights into the characteristics of the tumor microenvironment. Specifically, the ratio of CD8^+^ cells to CD11b^+^Ly6G^+^ cells (identified as a neutrophil population) was evaluated. In untreated control tumors, no significant differences in this ratio were observed, although the ratio in T-cell–low tumors tended to be lower than in T-cell–high and MC38 tumors (Figure 7).

Following treatment at a fluence rate of 116 mW·cm^−2^, the ratio increased in both T-cell–high and MC38 tumors compared to the corresponding control tumors. In contrast, the ratio in T-cell–low tumors remained low and was significantly lower than the ratios observed after treatment in T-cell–high and MC38 tumors (0.1 ± 0.1 vs. 1.5 ± 1.2 and 1.4 ± 0.7 with *p* ≤ 0.01 and *p* ≤ 0.05, respectively). These findings suggest that the tumor microenvironment in T-cell–high and MC38 tumors is more supportive of an anti-tumor immune response.

Treatment at the lower fluence rate of 50 mW·cm^−2^ also increased the ratio in T-cell–high tumors compared to untreated controls, whereas no increase was observed in T-cell–low tumors. Again, the post-treatment ratios differed significantly between these tumor types (*p* ≤ 0.05). This indicates that reducing the fluence rate does not compromise the favorable immune cell balance in T-cell–high tumors and may therefore preserve a more effective anti-tumor immune response.

Moreover, an overall upward trend in the ratio was observed in MC38 and T-cell–high tumors, suggesting that PDT delivered at either 116 or 50 mW·cm^−2^ promotes remodeling of the immune microenvironment in these tumor types.

## 3. Discussion

The tumor microenvironment of different tumor types plays a crucial role in the efficacy of PDT and the induction of immune responses. In this study, we aimed to investigate if different PDT regimens influence the treatment outcomes in pancreatic cancer models characterized by varying levels of T-cell infiltrations [26], and a colon cancer model (MC38), which we previously demonstrated to be broadly responsive to the combination of PDT and immunotherapy [25].

Notably, the MC38 model exhibited an initial higher blood volume fraction compared to the PDAC tumors, which can be attributed to better delivery and distribution of the photosensitizer within the tissue [2]. This suggests that the MC38 model may be more amenable to PDT treatment due to enhanced photosensitizer delivery and distribution. However, Figure 3 presents fluorescent data indicating that the initial fluorescent signal of Bremachlorin (a measure of the photosensitizer’s concentration prior to PDT) does not differ significantly between T-cell–high, T-cell–low, and MC38 tumors. In fact, T-cell–low tumors exhibited a higher amount of fluorescence compared to MC38 tumors. This suggests that the bioavailability and distribution of Bremachlorin are similar across tumor types, and that MC38 tumors do not necessarily take up more Bremachlorin than the PDAC tumor types.

Our results demonstrate low blood saturation in PDAC tumors independent of reduced blood volume fractions, suggesting high oxygen consumption in these tumors. Koong et al. reported regions of low oxygenation and hypoxia in human PDAC tumors [17]. They measured the tissue oxygenation in PDAC tumors and adjacent normal pancreas tissue intraoperatively along two to three measurement tracks using Eppendorf pO_2_ histography, a needle-electrode-based technique. Extremely low oxygen tensions were observed in 24% to 94% of measurements across seven patients, highlighting pronounced spatial and inter-patient heterogeneity in tumor oxygenation. Similarly, we observed substantial variability in baseline StO_2_ values between tumors (Figure 4). As the optical fiber probe we employed in our study interrogates only a relative small tissue volume, local oxygenation heterogeneity within the tumor is likely to have contributed to the observed differences in baseline StO_2_. While this explanation is particularly relevant for the highly heterogeneous PDAC microenvironment, a similar effect may also account for the variability observed in the MC38 tumors.

These observed physiological conditions pose a substantial challenge to oxygen-dependent modalities, including photodynamic therapy (PDT) [18]. Beyond hypoxia, PDAC is characterized by a dense desmoplastic stroma consisting of extracellular matrix proteins, activated fibroblasts, and compressed vasculature. Recent reviews on PDT for pancreatic cancer have emphasized that this desmoplastic microenvironment can impede photosensitizer delivery, restrict oxygen diffusion, and limit light penetration, thereby reducing PDT efficacy and contributing to treatment resistance [28]. Furthermore, emerging strategies in the PDT field increasingly focus on overcoming stromal and hypoxic barriers through targeted photosensitizer delivery systems, oxygen-generating nanoplatforms, and microenvironment-modulating approaches, underscoring the recognized clinical importance of these limitations [29]. Despite growing recognition of the importance of the PDAC microenvironment, relatively little is known about the real-time physiological response of PDAC tumors to PDT, particularly with respect to the balance between photochemical oxygen consumption and oxygen resupply within the highly hypoxic PDAC microenvironment during treatment. Therefore, one of the goals of this study was to determine whether lowering the fluence rate would reduce the consumption of oxygen during PDT, thereby potentially improving treatment outcomes. However, we observed no significant differences in blood oxygen saturation and blood volume fraction during PDT when the fluence rate was reduced from high (116 mW·cm^−2^) to a moderate fluence rate (50 mW·cm^−2^). This suggests that further lowering the fluence rate may be necessary to achieve the desired effect of increasing efficacy.

Bremachlorin is a hydrophilic mixture of 3 photosensitizers, as follows: chlorin e6 (≈80%), chlorin p6 (≈15%), and purpurin 5 (≈5%). While chlorin e6 is the predominant component of Bremachlorin, we have previously demonstrated through spectral analysis that the fluorescence kinetics of Bremachlorin differ to those of chlorin e6 [23]. It is likely that the photobleaching kinetics of each component are different and have a different relationship to the deposition of reactive oxygen in tissue and in turn therapeutic efficacy. Therefore, mechanistic conclusions based on differences in photobleaching kinetics of Bremachlorin should be interpreted with care. Notwithstanding these considerations, in general, photobleaching was more pronounced in the MC38 tumors than in either of the two PDAC models, consistent with the higher baseline StO_2_ levels observed in this tumor type. In the MC38 tumors, illumination with a lower fluence rate resulted in a greater extent of photobleaching, consistent with our previous observations and reports by others for a variety of photosensitizers [3,30,31]. The inter-tumor variability observed in baseline StO_2_ levels is also evident during PDT, especially for the PDAC tumors. Notably, the magnitude of the decrease in StO_2_ did not consistently correlate with the extent of photobleaching, even though both were assessed within the same localized tumor region. Several response patterns were observed. In the majority of MC38 tumors, the relationship was relatively straightforward, as follows: blood oxygen saturation remained stable or decreased only modestly, while pronounced photobleaching occurred. This suggests that local oxygen availability was sufficient to support the photodynamic reaction without substantially compromising vascular oxygenation, which was even clearer for the lower fluence rate group. In contrast, most PDAC tumors exhibited limited photobleaching accompanied by low baseline StO_2_ levels and/or a marked decline in StO_2_ during treatment. These findings indicate restricted local oxygen availability, where even oxygen replenishment from the vasculature may have been insufficient to sustain an effective photodynamic response. Only in two mice with a PDAC tumor, the level of photobleaching was almost complete and combined with a blood saturation level higher than 20% during the whole treatment. This finding indicates the presence of a tumor blood oxygen saturation threshold, whereby photobleaching is observed only when the saturation level exceeds a sufficiently high value.

Notably, both T-cell–low and MC38 tumors showed a significant reduction in blood volume fraction at both high and moderate fluence rates, while T-cell–high tumors showed a reduction in blood volume fraction only at the moderate fluence rate. This effect could be beneficial, as it can lead to the destruction of tumor micro-vessels, thereby depriving the tumor of essential nutrients. However, it can also result in temporary vasoconstriction, causing hypoxia and potentially compromising treatment efficacy [30].

Collagen type I is one of the main proteins making up PDAC’s stroma [32]. The presence of this stroma is thought to be the major contributor to the resistance of PDAC to therapeutic interventions [32]. However, reducing type I collagen has been shown to accelerate the progression of pancreatic cancer [33], highlighting the complex role of the tumor stroma in cancer development. While the stroma presents an attractive therapeutic target, studies have shown that complete removal of the stroma can paradoxically lead to increased disease progression and decreased survival rates [34]. Current strategies for targeting the stroma focus on modifying its density, rather than eliminating it [34]. In our study, we observed a significant reduction in collagen I levels 10 days after PDT at both fluence rates in both PDAC tumor types. This finding is consistent with previous research, where similar reductions in collagen I have been observed following PDT treatment [28,35]. The observed reduction in collagen I expression may be the result of, at least in part, PDT-induced effects on cancer-associated fibroblasts (CAFs) within the PDAC tumor microenvironment. Chlorin e6, a component of Bremachlorin, has been reported to accumulate in CAFs, rendering these cells susceptible to photodynamic damage [36]. Although the present study did not directly assess CAF viability or function following PDT, it is conceivable that a reduction in the viable CAF population contributes to decreased collagen I production and secretion, thereby leading to the lower collagen I levels observed after treatment. The reduction in collagen I could improve drug delivery and therapeutic response, but may also influence the tumor microenvironment and disease progression. Further investigation is needed to fully understand this effect.

Notably, T-cell–high tumors treated with PDT at 116 mW·cm^−2^ or 50 mW·cm^−2^ to 116 J·cm^−2^, showed a significant reduction in proliferating tumor cells compared to their respective controls (Figure 5c). This suggests that PDT can effectively reduce the number of proliferating cancer cells in PDAC tumors with high T-cell infiltration.

The analysis of pan keratin^+^ PD-L1^+^ cells in all three tumor types showed no significant difference in expression between control groups and those treated with high fluence rate and moderate fluence rate PDT. However, the expression of pan keratin^+^ PD-L1^+^ cells in MC38 tumors was significantly higher (Figure 6e). This elevated expression of PD-L1 in MC38 tumors suggests that these tumors may be more responsive to checkpoint blockade therapies that target the PD-L1 pathway, which is consistent with previous observations [37,38,39].

Previous studies have suggested that PDT can destroy lymphocytes, particularly activated lymphocytes [10]. Previously, we also showed this in the T-cell–high tumors treated with 116 mW·cm^−2^ and tissues collected after full regrowth to 500% of the treatment volume [27]. However, in the current study, we found that the level of CD8^+^ T-cells did not decrease significantly in the PDT treated tissues (Figure 6d). In fact, in the T-cell–high tumors we observed an increase in CD8^+^ cells 10 days after both fluence rate PDT’s, which was statistically significant for the moderate fluence rate group. This finding suggests that PDT delivered at moderate fluence rates (50 mW·cm^−2^) may exert distinct immunomodulatory effects within the PDAC tumor microenvironment. Further investigation into the type of CD8^+^ T-cells present, such as exhausted, active, or memory T-cells, is necessary to fully understand the implications of this observation and the potential immunomodulatory effects of PDT at moderate fluence rates. The increase in CD8^+^ cells 10 days after PDT was not detected in the T-cell–low tumors, despite the identical PDT treatment conditions. This finding suggests that the tumor microenvironment of these tumors may prevent or limit the infiltration of CD8^+^ T cells, thereby constraining the development of an effective PDT-induced antitumor immune response. It is known that stromal barriers, suppressive myeloid populations, abnormal vasculature, TGF-β signaling, and hypoxia can prevent CD8+ T cells from entering tumors even when antitumor immune responses are generated by in our case PDT [40,41,42]. However, repeated PDT treatment may succeed in inducing a more potent anti-tumor adaptive immune response and a favorable TME remodeling.

Consistent with our previous studies, we found that T-cell–high control tumors exhibited a significantly higher quantity of CD8^+^ T-cells in comparison to T-cell–low tumors (Figure 6d) [43,44]. PDT treatment led to a significant increase in CD8^+^ cells in those T-cell–high tumors. This increase in CD8^+^ cells was not observed for MC38 tumors whereas those tumors showed a significant reduction in tumor volume, highlighting the importance of characterizing the T-cell type and suggesting that CD8^+^ T-cells may not be the sole indicator of a favorable response. When examining the ratio of CD8^+^ cells to CD11b^+^Ly6G^+^ (neutrophils), we observed that T-cell–high tumors had a higher ratio of CD8^+^ to neutrophils in comparison to T-cell–low PDT-treated samples (Figure 7). Achieving a favorable balance between tumor-fighting immune cells and tumor-promoting immune cells is critical for improving response rates [45]. A higher ratio of CD8^+^ T-cells to neutrophils may be indicative of a more effective anti-tumor immune response.

Our previous study demonstrated that combining PDT with the checkpoint blocker anti-PD-L1, at a fluence rate of 116 mW·cm^−2^ to 116 J·cm^−2^, resulted in an additive effect in T-cell–high PDAC tumors [27]. However, in T-cell–low tumors, the baseline level of CD8^+^ T-cells is likely insufficient to achieve a significant improvement with anti-PD-L1 treatment, making it challenging to shift the balance between tumor-fighting and tumor-promoting immune cells in favor of an anti-tumor response. Interestingly, Lobo et al. 2023 found that redaporfin-mediated PDT increased the expression of CD8^+^, CTLA-4, and PD-L1 in B16F10 and 4T1 tumor types [46]. In contrast, MC38 tumors showed no increase in PD-L1 expression after PDT, although they consistently expressed high levels of PD-L1 across all conditions. Investigating CD8^+^ expression in PDAC tumors and its interaction with CTLA-4 could provide valuable insights into a potential combination treatment for PDAC, especially since our previous study showed that both T-cell–high and T-cell–low PDAC tumor types express T regulatory cells [44]. However, a study by Bengsch et al. 2017 found that CTLA-4 is not the primary regulator responsible for T-cell blocking in PDAC, which may be attributed to the low antigenicity of PDAC tumors [47]. The authors suggested that targeting CD40, a member of the tumor necrosis factor (TNF) family, could enhance T-cell response by increasing antigenicity as well as activating antigen-presenting cells [47,48]. Nonetheless, their findings indicated that CD40 agonists alone are insufficient to induce an anti-tumor immune response. This suggests that combining CD40 agonists with PDT may be a promising strategy, since both treatments have been shown to alter the tumor stroma as well as to modify the immune landscape in PDAC.

Our study on the effect of PDT on the tumor microenvironment in various preclinical tumor models has revealed that PDT is able to affect the TME as demonstrated by the reduction in collagen type I and by shifting the immune cell landscape toward a less immunosuppressive type. Notably, our findings suggest that PDT can modulate the TME, creating a more favorable environment for anti-tumor immune responses. Interestingly, the moderate fluence rate used in our study did not improve tumor regression but showed similar influences on the TME compared to the high fluence rate. However, it did have a significant impact on the immune cells’ population, particularly in T-cell–high tumors, where it significantly increased the number of CD8^+^ T-cells. This suggests that the moderate fluence rate may have distinct immunomodulatory effects on the immune cell landscape, highlighting the importance of further investigation into the optimal PDT parameters for tumor treatment.

## 4. Materials and Methods

### 4.1. Animal Model

Female C57BL/6 (strain C57BL/6JOlaHsd, RRID: IMSR_ENV:HSD-057) mice were purchased from Inotiv (Horst, The Netherlands). All animal experiments were approved by the Institutional Animal Care and Bioethics Committee of the Erasmus MC, Rotterdam, The Netherlands (approval number AVD1010020209868 and work protocol numbers SP2300064 and SP2300250). Experiments were conducted in accordance with the national guidelines and regulations established by the Dutch Experiments on Animals Act (WoD) and by the European Directive (2010/63/EU) on the Protection of Animals used for scientific purposes [49, 50].

### 4.2. Tumor Cell Lines

Murine pancreatic tumor cells clone types 2838c3 (referred to as T-cell–high, RRID: CVCL_YM18) and 6694c2 (referred to as T-cell–low, RRID: CVCL_YM31) were derived from tumor-bearing female KPC/Y mice [26]. These cells were provided by Dr. Ben Stanger (University of Pennsylvania). Murine colon adenocarcinoma MC38 cells were gifted from Prof. F. Ossendorp laboratory (LUMC, Leiden, The Netherlands). All three cell lines were cultured in high glucose Dulbecco’s Modified Eagle Medium (DMEM, Fisher Scientific BV, Landsmeer, The Netherlands), supplemented with 10% fetal bovine serum (FBS), and 1% penicillin-streptomycin at 37 °C in a humidified 5% CO_2_ incubator. Cells were regularly tested for mycoplasma.

### 4.3. Tumor Cell Injections and Measuring

Murine pancreatic tumor cells 2838c3 (T-cell–high) and 6694c2 (T-cell–low) (30 µL of 3 × 10^5^ PBS) and MC38 (100 µL 5 × 10^5^ PBS) were bilaterally injected subcutaneously into 6- to 8-week-old female C57BL/6 mice under general anesthesia (2.5% isoflurane/O_2_). Tumor growth was measured with digital calipers once the tumors became palpable. The three orthogonal diameters of the tumor were measured 3–5 times per week and the tumor volume was calculated using the formula Vt = D1 × D2 × D3 × PI/6. Experiments began when animals bearing murine pancreatic tumors from 2838c3 (T-cell–high) and 6694c2 (T-cell–low) clones and MC38’s first tumor reached approximately 60–80 mm^3^ (normalized to 100%).

### 4.4. Preparation and Storage of Bremachlorin

Photosensitizer Bremachlorin (Radapharma International B.V., Lelystad, The Netherlands) was divided into aliquots and stored at 4 °C. It was protected from light when handled. Bremachlorin was administered at 20 mg/kg, 3.5 mg/mL via intravenous (i.v.) injection into the tail vein.

### 4.5. In Vivo PDT Illumination

Experiments began with randomly assigning tumor-bearing animals to one of the treatment groups (*n* = 4 per group), as follows: control, PDT with 116 mW·cm^−2^ at 116 J·cm^−2^ (referred to as high fluence rate), or PDT with 50 mW·cm^−2^ at 116 J·cm^−2^ (referred to as moderate fluence rate).

Prior to PDT, tumor-bearing animals that received treatment were administered Bremachlorin and pain relief medication and were then kept in subdued light conditions for 6 h, as described previously [27]. Illumination was performed under gas anesthesia. Just before illumination, the skin overlying the tumor was loosely stretched using two sutures, and the quadfurcated fiber, for collecting the reflectance and fluorescence spectra, was placed underneath the skin, touching (but not pressing against) the tumor. Reflectance and fluorescence spectra were recorded before, during, and up to a maximum of 5 min after illumination, without repositioning the fiber. The illumination was delivered using a 662 nm diode laser (Newport Spectra−Physics B.V., Utrecht, The Netherlands) and a frontal light distributor (Medlight SA, Ecublens, Switzerland) with a dose of 116 J·cm^−2^ at a fluence rate of either 116 or 50 mW·cm^−2^ (*n* = 4 in each group).

All animals were monitored for 10 days after the experiment began, then sacrificed via cervical dislocation.

### 4.6. Monitoring PDT Using Single Fiber Reflectance and Fluorescence Spectroscopy

The experimental setup used to collect reflectance and fluorescence spectra is based on what has been described in detail previously [51]. In short, the setup utilizes a single optical fiber connected to a quadfurcated optical fiber, which has been polished at an angle of 15° to minimize internal specular reflections at the fiber tip. White light reflectance spectra were obtained with a halogen light source HL-2000-FHSA (Ocean Optics, Duiven, The Netherlands) via one arm and collection of the reflected light via a second arm connected to a spectrophotometer SD-2000 (Ocean Optics, Duiven, The Netherlands). A calibration procedure was performed to account for internal reflections, variability in lamp-specific output, and in fiber-specific transmission properties [52]. Fluorescence spectra were obtained immediately after the reflection measurement using excitation light split from the 662 nm therapeutic laser (at a fluence rate that approximately matched the treatment fluence rate in each group) connected to the third arm of the quadfurcated fiber. The emitted fluorescence was collected through the fourth arm of the quadfurcated fiber, filtered through the 670 LP filter, and connected to a second spectrograph (QE-6500, Ocean Optics, Duiven, The Netherlands).

### 4.7. Mathematical Analysis of Spectra

Single fiber reflectance spectra were analyzed using an analytical model designed to describe the wavelength-dependent optical properties, allowing for the extraction of physiological and morphological information from the sampled tissue, as previously outlined [53]. In brief, the attenuation resulting from absorption within the tissue was modeled using a modified Beer–Lambert law, which depends on both the tissue absorption coefficient (*μ_a_*) and the photon path length of the single fiber. The reflectance amplitude and the photon path length are influenced by the tissue’s scattering properties, specifically through the reduced scattering coefficient (*μ’_s_*) and the angular distribution of scattering (phase function).

The sensitivity of single-fiber reflectance signals to the phase function parameter gamma (*γ*) arises from the overlapping source–detector area inherent to single-fiber measurements, where the diffusion approximation is invalid [54]. Model fitting incorporated the fiber core diameter, the numerical aperture of the fiber, and the refractive index of the investigated solid tumor. The reduced scattering coefficient was modeled as a power-law function, with *γ* assumed to be constant across the fitted wavelength spectrum [52]. Rather than treating *γ* as a free fitting parameter, we fixed its value at 1.4, 1.6, and 1.8—a range representative of biological tissues [55]. We confirmed that this approach yielded estimates for the absorption coefficient within 6% of those obtained using alternative fitting methods.

We employed a Levenberg–Marquardt algorithm to estimate parameter values for microvascular haemoglobin oxygen saturation (StO_2_), blood volume fraction (BVF), and average blood vessel diameter (VD) by minimizing the chi-squared metric between the measured reflectance data and the model predictions. Confidence intervals for parameter estimates were calculated as the square root of the diagonal elements of the covariance matrix [56]. The parameter values were averaged over multiple measurements, weighted by the confidence intervals of individual spectral fits, and reported with the corresponding weighted standard deviation. Fluorescence spectra from all individual measurements were corrected for absorption and scattering as described previously [23]. Spectral deconvolution was utilized to separate the corrected fluorescence spectrum into components of auto-fluorescence and photosensitizer fluorescence. The subtraction of the auto-fluorescence spectrum yields the intrinsic fluorescence spectrum. The resulting intrinsic photosensitizer fluorescence spectrum was related to the product of the photosensitizer absorption coefficient (*μ_a_^f^* in mm^−1^) and the quantum yield (Q^f^) integrated over the wavelength. A basis spectrum for Bremachlorin was constructed from averaging three independent measurements taken from different mice, which subsequently served as a reference for fitting the intrinsic fluorescence spectrum. From this fitted curve, we determined the intrinsic fluorescence intensity and the associated 95% confidence interval, providing a robust estimate of the fluorescence signal.

### 4.8. Immunohistochemistry Staining

Tumors were harvested and fixed in 4% formalin followed by paraffin embedding. The formalin fixed paraffin embedded (FFPE) tissues were sliced into sections of 4 µm width and mounted on adhesive slides. For multi-immunohistochemistry staining, the primary antibodies used are displayed in Table 1. ZEISS Axioscan 7 fluorescence whole slide scanner (ZEISS, Oberkochen, Germany) at 20× magnification was used to scan all antibody-stained tissue slides. Consequent sections were stained with hematoxylin and eosin (H&E). H&E-stained sections were imaged on a NanoZoomer (Hamamatsu, Japan).

### 4.9. Fluorescence Imaging and Histology Analysis

Histological assessments were performed on H&E-stained tissue sections, which were blindly annotated by a pathologist to identify the tumor and necrotic areas of the tissue using NDP.view2 software (version 2.10.1, Hamamatsu, RRID: SCR_025177). The annotated sections were then used as a reference for the analysis of the fluorescent images, which were conducted using QuPath version 0.5.1 (RRID: SCR_018257) [57]. Whole tumor slices were analyzed to quantify the number of positively stained cells. In brief, the process involved manually outlining the tumor and necrotic tissue areas based on the pathologist’s H&E annotations, while also removing any staining, tissue artifacts, or necrotic areas to ensure accurate results. Next, nuclear segmentation was applied to individual cells, and the cell type was determined based on the maximum intensity of a threshold value. The number of positively stained cells was then calculated per square millimeter, enabling a precise quantification of the tumor’s characteristics.

### 4.10. Statistical Analysis

All data are presented as mean ± standard deviation (SD) or as mentioned, with the exception of data collected using single fiber spectroscopy. For the single fiber spectroscopy, the fitted values for StO_2_, BVF, VD, and Q.*μ_a_^f^* were weighted by the confidence interval to result in a weighted mean ± SD. Statistical significance was determined using the following methods: Student’s *t*-test/Analysis of Variance (ANOVA)/SNK multivariate analysis and Chi quadrant analysis. The ratio of CD8^+^ to CD11b^+^Ly6G^+^ was calculated using the individual tumor-level ratios before averaging per group.

For all data, differences were considered statistically significant when *p* ≤ 0.05. A Two-way ANOVA with Boneferroni’s post hoc test for multiple comparisons was used to compare groups.

All statistical analysis and graphing were performed using GraphPad Prism version 10.4.1 and 10.6.1.

## 5. Conclusions

Our study of the effects of PDT on the tumor microenvironment in various pre-clinical tumor models has revealed that PDT is able to affect the TME as demonstrated by the reduction in Collagen type I and by shifting the immune cell landscape toward a less immunosuppressive type. Bremachlorin-PDT has a significant impact on the immune cell population, particularly in T-cell–high tumors, where it significantly increased the number of CD8^+^ T-cells. This suggests that PDT has distinct immunomodulatory effects on the immune cell landscape, highlighting the importance of further investigation into the optimal PDT parameters for tumor treatment.

## Figures and Tables

**Table 1:** Antibodies for Immunohistochemistry.

Antibody	Type	Source	Clone	Con/dil	Cat#	RRID
Calreticulin	Anti-Rabbit	Novus	Polyclonal	1/100	I-7	
CD8	Anti-Rabbit	Abcam	Monoclonal	1/100	AB217344	AB_2890649
CD11b	Anti-Rabbit	Abcam	Monoclonal	1/200	AB133357	AB_2650514
Collagen I	Anti-Rabbit	Abcam	Monoclonal	1/3000	AB138492	AB_2861258
HIF-1α	Anti-Rabbit	Genetex	Polyclonal	1/400	GTX30647	AB_625919
Ki-67	Anti-Rabbit	Ventana	30-9	2 µL/mL	790-4286	AB_2631262
Ly6G	Anti-Rabbit	Abcam	Monoclonal	1/100	AB238132	AB_2923218
Pan Keratin	Anti-Mouse	Ventana	AE1/AE3/PCK26	46.3 µL/mL	760-2135	AB_2810237
PD-L1	Anti-Rabbit	Ventana	SP263	1.61 µL/mL	741-4905	/

## Data Availability

The original data presented in the study are openly available in Zenodo at https://doi.org/10.5281/zenodo.21408798 accessed on the 7 October 2026.

## Supplementary Materials

The following supporting information can be downloaded at: https://www.mdpi.com/article/10.3390/ijms27209374/s1.

## Abbreviations

The following abbreviations are used in this manuscript:

PDT	Photodynamic therapy
PDAC	Pancreatic ductal adenocarcinoma
TME	Tumor microenvironment
ROS	Reactive oxygen species
