# Supplementary material for: Bremachlorin-Based Photodynamic-Therapy-Induced Effects in Low-Oxygenated Pancreatic Ductal Adenocarcinoma (PDAC) Tumor Microenvironment
Source: Int J Mol Sci. 2026 Oct 21;27(20):9374. doi: 10.3390/ijms27209374 (PMC13705006; doi:10.3390/ijms27209374)
Supplement: Supplementary file 1 [file ijms-27-09374-s001.zip › ijms-4470536-supplementary.pdf]

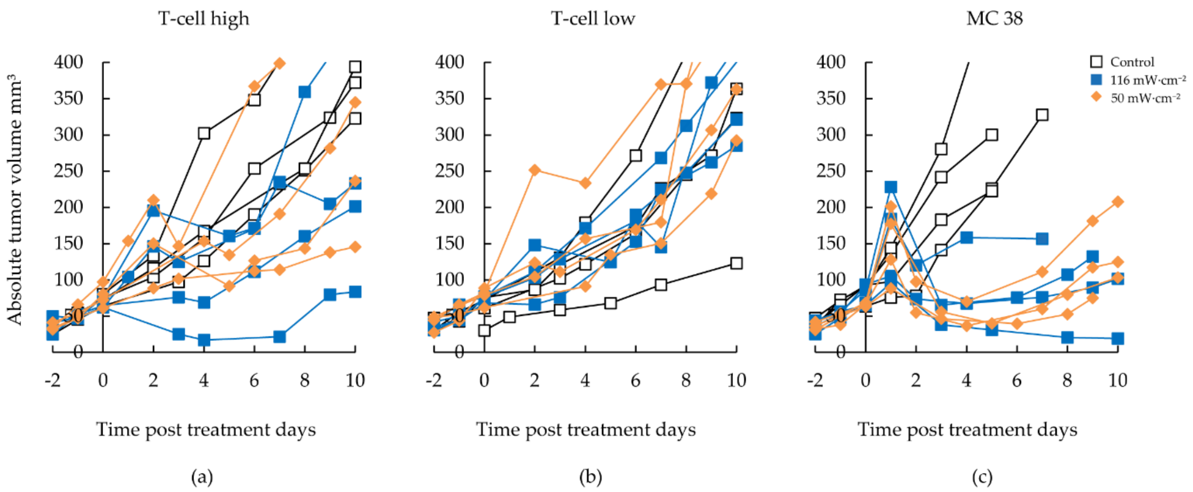

Supplementary Figure S1: Individual absolute tumor growth curves of: (a) T-cell-high PDAC; (b) T-cell-low PDAC and (c) MC38 tumors for control (black outlined squares) or after Bremachlorin PDT to 116 J·cm^{-2} at 116 mW·cm^{-2} (blue filled squares) or at 50 mW·cm^{-2} (orange filled diamonds).

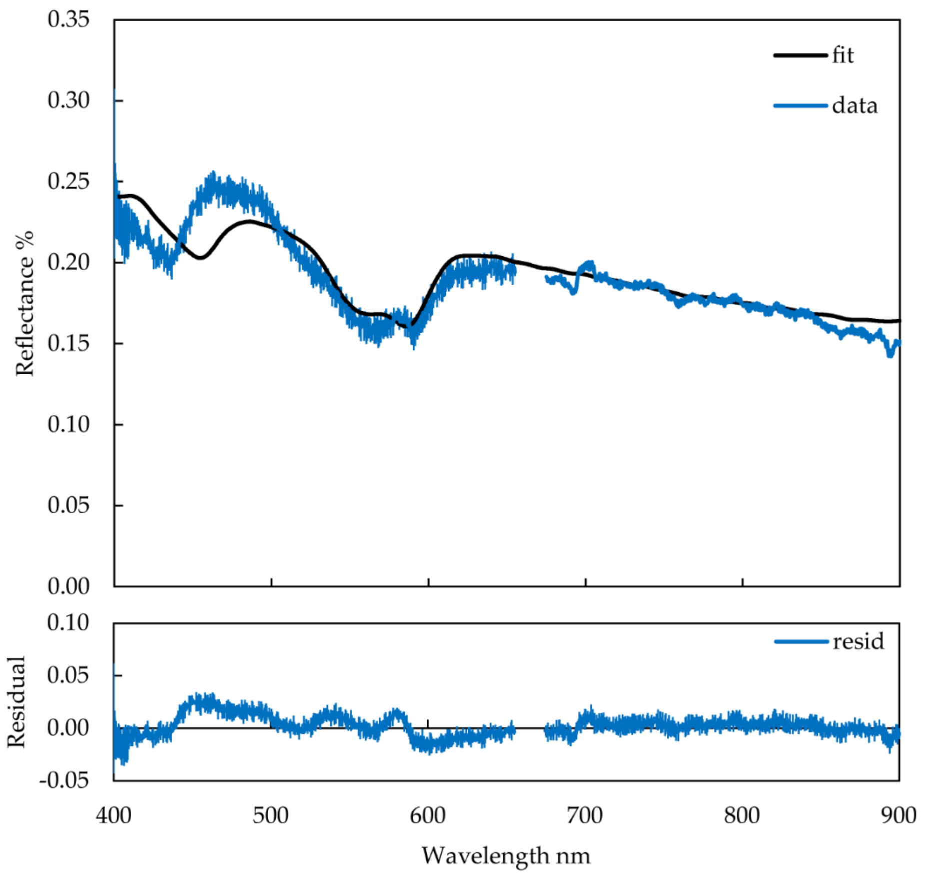

Supplementary Figure S2: Representative reflectance spectrum (red) and corresponding fitted spectrum (black) with the residual spectrum per wavelength in tumor tissue. Note: data points surrounding the PDT laser illumination wavelength were not included in the analysis. The goodness of fit is reflected in the confidence intervals of the fitted parameters.

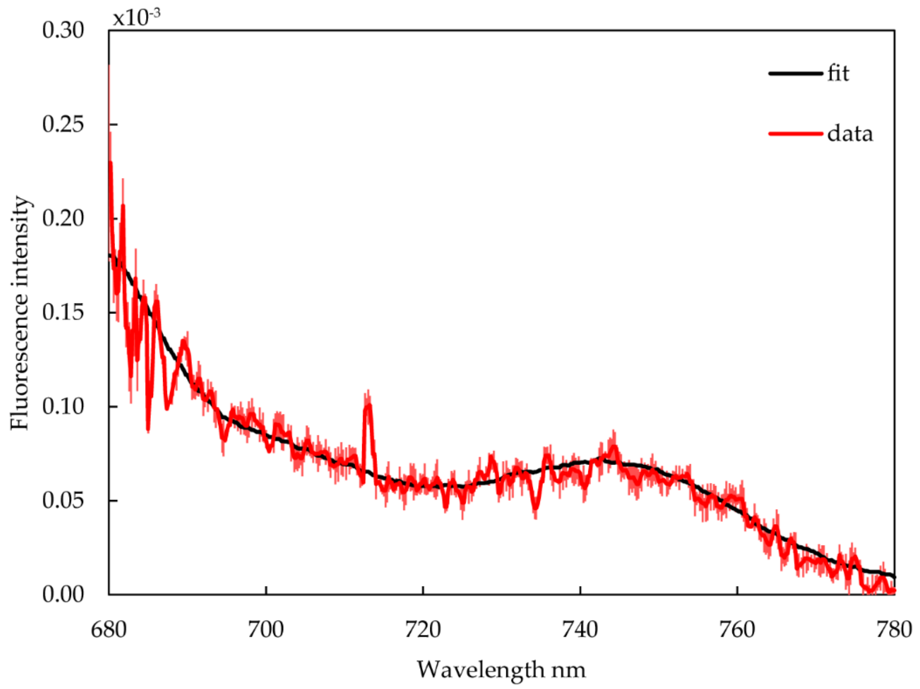

Supplementary Figure S3: Representative fluorescence spectrum (red) and corresponding fitted spectrum (black) of Bremachlorin in tumor tissue. The goodness of fit is reflected in the confidence intervals of the fitted parameters.

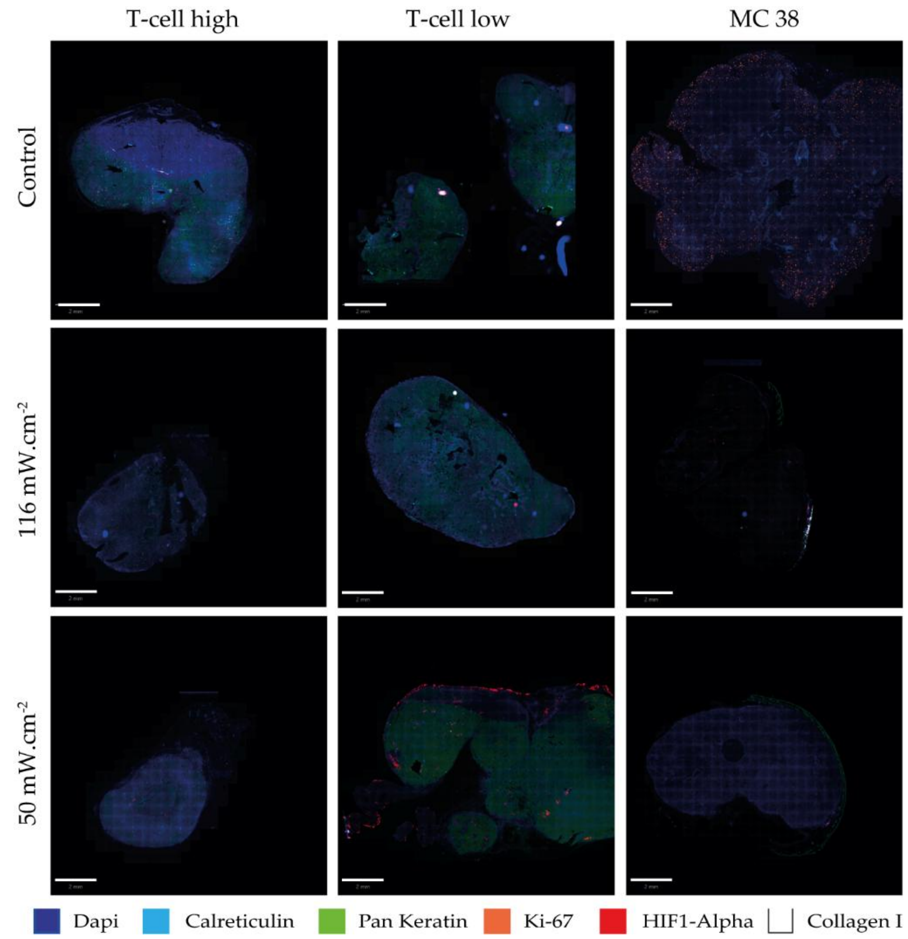

Supplementary Figure S4: Representative multiplexed fluorescent images of T-cell high, T-cell low and MC38 whole tumor tissue sections from untreated controls and Bremachlorin PDT-treated (116 $J.cm^{-2}$ at 116 $mW.cm^{-2}$ or 50 $mW.cm^{-2}$) stained for DAPI, calreticulin, pan keratin, Ki-67, HIF1 alpha and collagen type I. Bar is 2 mm.

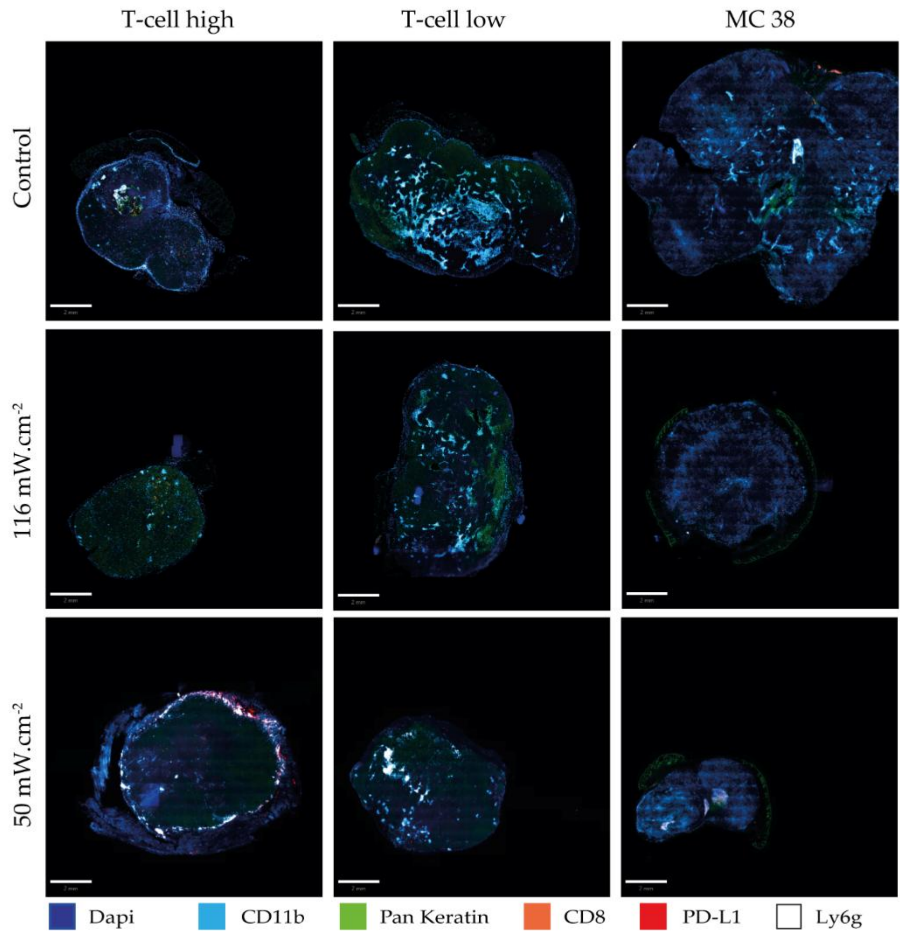

Supplementary Figure S5: Representative multiplexed fluorescent images of T-cell high, T-cell low and MC38 whole tumor tissue sections from untreated controls and Bremachlorin PDT-treated (116 J.cm^{-2} at 116 mW.cm^{-2} or 50 mW.cm^{-2}) stained for DAPI, CD11b, pan keratin, CD8, PD-L1 and Ly6g. Bar is 2 mm.
